# Temozolomide-associated blepharoconjunctivitis: a case report

**DOI:** 10.1186/s12886-024-03417-6

**Published:** 2024-04-12

**Authors:** Tom Kornhauser, John D Pemberton

**Affiliations:** Jones Eye Institute, University of Arkansas for Medical Sciences, 4301 W. Markham Street, 72207 Little Rock, AR USA

**Keywords:** Temozolomide, Glioblastoma multiforme, Blepharoconjunctivitis, Blepharitis, Case report

## Abstract

**Background:**

Temozolomide (TMZ) is an effective oral alkylating agent used in treating glioblastoma multiforme (GBM) and high-grade gliomas. It works by introducing methyl groups into DNA, inhibiting cell division. A case of blepharoconjunctivitis linked to the administration of TMZ is detailed in this report.

**Case presentation:**

We present a case of a 58-year-old African-American man diagnosed with GBM. Following adjuvant TMZ treatment, he developed blepharoconjunctivitis, characterized by eyelid and conjunctival inflammation. Symptoms included eyelid swelling, crusting, and conjunctival discharge, which were promptly resolved with topical steroid cream and eye drops.

**Conclusions:**

Reports specifically linking TMZ to blepharoconjunctivitis are limited. The exact mechanism remains unclear but may involve inflammation extending from blepharitis to the conjunctiva. Healthcare providers must recognize and manage ophthalmic complications promptly. This case report highlights blepharoconjunctivitis associated with TMZ use in a GBM patient. While TMZ is an effective treatment, ophthalmic side effects can occur.

## Background

Temozolomide (TMZ) is an oral alkylating agent with an excellent central nervous system penetration, that has been used in the treatment of gliomas, melanoma, pituitary tumors, and primary vitreoretinal lymphomas. TMZ works by adding methyl groups to DNA, thus preventing the cells from dividing [1–4]. For glioblastoma multiforme (GBM) tumors, TMZ is considered a highly effective primary therapy compared to using radiotherapy alone either as an adjuvant or concomitant treatment. This agent extends survival and progression-free survival (PFS) but with an increased risk of early adverse events. In recurrent high-grade gliomas, TMZ improves PFS and may have beneficial effects on quality of life [4]. 

The frequently reported adverse effects associated with the use of this agent encompass alopecia, nausea, vomiting, anorexia, headache, thrombocytopenia, anemia, liver enzyme irregularities, and constipation. Other less common ophthalmic and dermatological side effects are blurred vision, xeroderma, skin erythema, pruritus, rash, and disseminated superficial actinic porokeratosis [5–9]. To our knowledge, the case that we discuss in this paper is the first report of blepharoconjunctivitis associated with TMZ use.

## Case presentation

A 58-year-old African-American man came to our clinic for consultation about his eyelid swelling and periocular discharge. He described having several days of unrelenting eyelid swelling, eyelid crusting, and conjunctival discharge, with no accompanying itching. At the time of our initial exam, he had not had any specific treatment for the complaint. Six months prior to the onset of his complaints, he was diagnosed with a grade IV glioblastoma accompanied by isocitrate dehydrogenases (IDH) mutation and *O*^6^-methylguanine-DNA methyltransferase (MGMT) promoter methylation. Shortly after, he initiated intensity-modulated radiation therapy of 60 Gy in 2 Gy daily fractions with concurrent TMZ 165 mg per day for six weeks. The right forehead received a total of 20–25 Gy, and the medial periocular region received a total of 5 Gy. Four weeks after completing the initial TMZ treatment, he then started adjuvant TMZ treatment cycles: 330 mg per day for five days, with a four-week break between each cycle. His presenting complaint appeared twenty-four hours after initiating the latter TMZ cycle.

Previous ocular and other medical history were unremarkable, with no prior history of blepharitis-associated dry eye, hypercholesterolemia, or rosacea. His visual acuity was 20/20 in both eyes. The patient’s ocular motility and pupillary examinations were normal. The eyelid examination revealed significant periorbital edema, marked by swollen lid margins, crusting, and the formation of collarettes at the base of the lashes, along with occasional breaks and bleeding of the skin. Additionally, we saw a thick mucus discharge on the eyelid margins of both eyes. Both conjunctivae demonstrated follicular reaction of the palpebral conjunctiva and injection of the bulbar conjunctiva, with no evidence of allergy-related signs such as papillary reaction. Both corneas were clear with inferior punctate epithelial erosions. The rest of the exam of the anterior and posterior segments was unremarkable. The patient denied experiencing any rash or dermatological symptoms elsewhere on his body, except for the acute symptoms affecting the eyes. Based on the clinical history, symptoms, and clinical exam, including no obvious rosacea or systemic allergic reaction on general examination, we made a diagnosis of blepharoconjunctivitis.

**Fig. 1:**
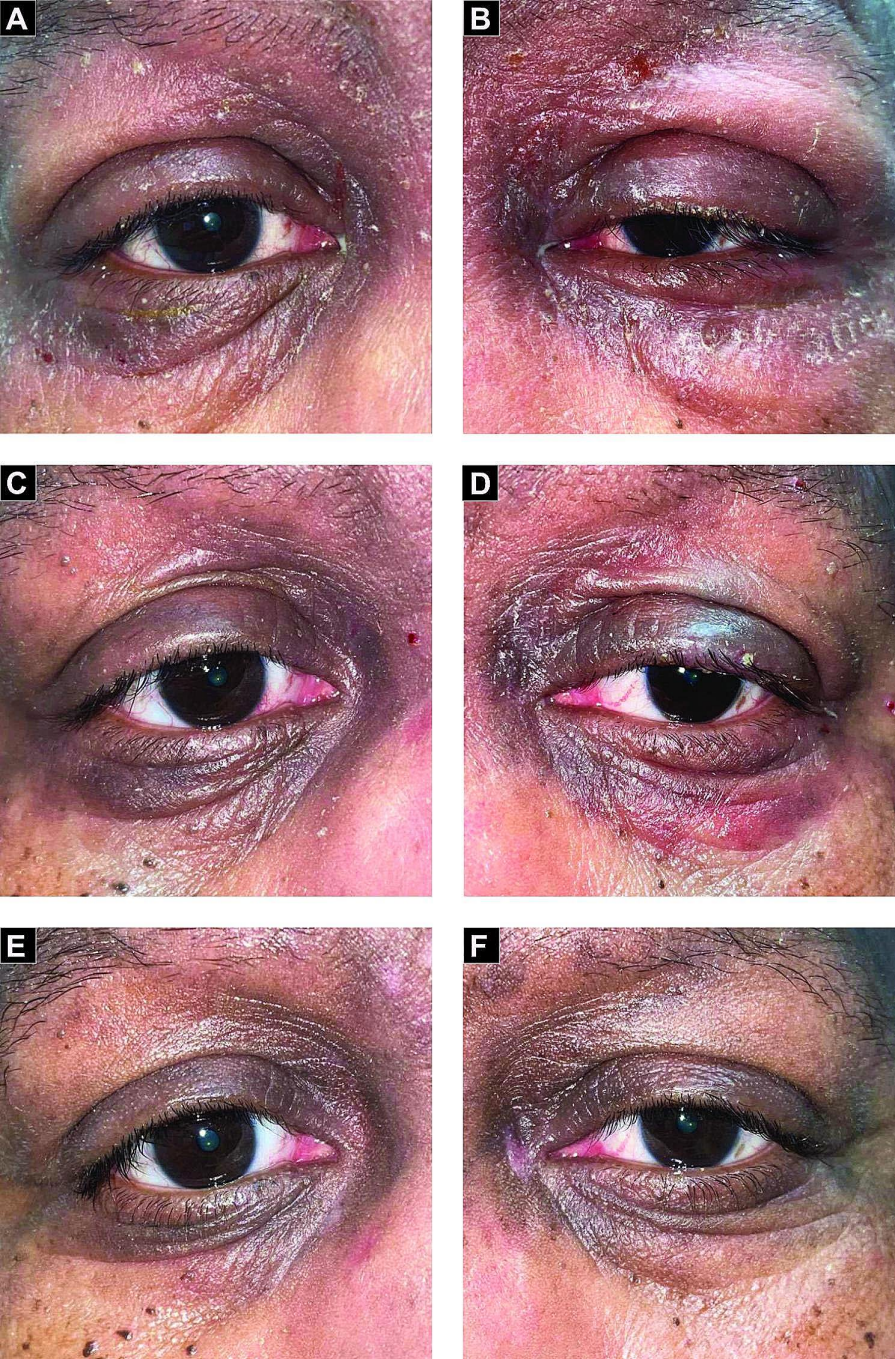
External photographs showing bilateral blepharoconjunctivitis with periorbital changes. **A** and **B** shows initial presentation of the right and left eye, respectively. **C** and **D** shows the immediate response to topical steroids on the right and left eye after 4 days, respectively. **E** and **F** shows a complete resolution of the right and left eyes after 3 weeks, respectively

We prescribed applying triamcinolone acetonide 0.1% topical cream twice-daily to the eyelids for ten days We also prescribed prednisolone acetate 1.0% eye drops four times a day for 10 days to treat the associated conjunctival findings. We performed repeated slit-lamp examinations on the patient at 4 days and again at 3 weeks. By the time of the last exam, all signs and symptoms had resolved (Fig. 1). The oncology team decided to discontinue TMZ both in response to the patient’s reaction and as the patient enrolled in a GBM trial.

## Discussion

TMZ can help treat several conditions and types of tumors. Gliomas are tumors from glial cells in the brain and spinal cord, classified by the World Health Organization into grades I-IV. Grades III and IV, high-grade gliomas (HGG), are aggressive, with GBM, anaplastic astrocytoma, and anaplastic oligodendrocytoma being common. HGG has an annual incidence under 8 per 100,000, comprising about 1% of new cancers. TMZ treatment is well-tolerated, causing 5–10% hematological toxicity. It offers a favorable median survival of around 16 months [4]. 

Blepharoconjunctivitis is an ophthalmic condition that encompasses both blepharitis and conjunctivitis. This condition is characterized by inflammation occurring at both the eyelid margin and the surrounding conjunctiva. Various underlying causes have been associated with blepharoconjunctivitis, which is frequently multifactorial in nature. Of note, certain drug adverse reactions have also been associated as the culprit of this condition [10–15]. In the process of differential diagnosis, potential causes were carefully considered based on the patient’s history and physical examination. Allergic blepharoconjunctivitis, typically accompanied by itching, a rash, or a papillary reaction of the conjunctiva, was ruled out due to their absence. The possibility of an isolated eyelid dermatitis, characterized by skin changes on the eyelid without conjunctival involvement, was excluded based on the presence of a follicular reaction observed during the examination. This reaction aligns more closely with a pattern consistent with blepharoconjunctivitis rather than isolated dermatitis with a spill that caused irritation. However, it is accurate to note that the patient did exhibit both eyelid dermatitis and conjunctivitis. The immediate onset of symptoms following TMZ administration, the absence of systemic signs of infection, and the resolution of symptoms upon discontinuation of the drug, strongly suggests that the blepharoconjunctivitis in this case is more likely related to TMZ use rather than stemming from an infectious cause. Unfortunately, swabbing was not performed to further exclude viral blepharoconjunctivitis, which could have provided additional clarity in diagnosis.

There have been no reports thus far linking TMZ with the occurrence of blepharoconjunctivitis. However, other alkylating agents, such as cyclophosphamide, ifosfamide, and busulfan have been associated with blepharoconjunctivitis. Alkylating agents are known to cause dermatological adverse reactions due to their ability to damage DNA and disrupt cellular function. These agents chemically modify DNA molecules, leading to the formation of DNA adducts and cross-links. This DNA damage can trigger various cellular responses, including inflammation, apoptosis, and impaired cell replication. In the skin, alkylating agents can directly affect the rapidly dividing cells of the epidermis and hair follicles. This can lead to a range of dermatological adverse reactions, including rash, dryness and redness of the skin, and pruritus [5–8]. The underlying mechanism and cause of conjunctivitis in these cases are still unclear. The conjunctiva is composed of non-keratinized squamous epithelial cells and is relatively resistant to the cytotoxic effects of most chemotherapeutic agents, including alkylating agents. However, the inflammation associated with blepharitis can extend to involve the adjacent conjunctiva, leading to the development of blepharoconjunctivitis.

In our patient, TMZ is essential for managing GBM. Nevertheless, the initiation of the third TMZ cycle resulted in significant blepharoconjunctivitis. One might speculate that radiation dermatitis could be the cause of the periocular flareup. However, acute radiation-induced skin changes typically occur within days and peak within approximately two weeks [16]. In our case, these changes occurred abruptly more than one month after the last radiation dose. Establishing a cause-and-effect relationship between the use of TMZ and the abrupt appearance of blepharoconjunctivitis can be challenging due to the potential involvement of multiple causes for this eye condition. There could be other factors at play including confounding variables and the occurrence of unrelated events during that period.

Managing blepharoconjunctivitis encompasses a range of therapeutic approaches. The resolution of most cases has been demonstrated using topical and oral antibiotics, as well as topical steroids. Additionally, techniques such as warm lid compressions and eyelid hygiene have been reported to provide relief from blepharoconjunctivitis by promoting the stimulation of meibomian glands [17]. We elected to use topical steroid cream and eye drops for treatment in this case. Symptoms tremendously improved four days after initiating treatment.

In summary, this case report highlights a unique occurrence of blepharoconjunctivitis associated with the use of TMZ in a patient with glioblastoma grade IV. While TMZ is effective in treating glioblastoma, it can lead to ophthalmic side effects. Alkylating agents like TMZ have been known to cause dermatological adverse reactions due to their DNA-damaging properties, but specific reports linking TMZ with blepharoconjunctivitis are limited. This case underscores the importance of considering potential ophthalmic complications and implementing appropriate management strategies when administering alkylating agents.

The management of blepharoconjunctivitis involves various therapeutic approaches. Prompt treatment with topical steroid cream and eye drops resulted in significant symptom improvement in the reported case. However, further research is needed to better understand the mechanism and incidence of blepharoconjunctivitis associated with alkylating agents like TMZ. To establish a definitive cause, further investigations and well-controlled studies are necessary to rule out other potential contributors and demonstrate a consistent pattern of association. Healthcare providers should remain vigilant for potential ophthalmic side effects in patients undergoing treatment with these agents, ensuring comprehensive care to optimize treatment outcomes and patient well-being.

## Data Availability

The datasets used and/or analysed during the current study are available from the corresponding author on reasonable request.

## Abbreviations

GBM: Glioblastoma multiforme
HGG: High-grade gliomas
IDH: Isocitrate dehydrogenases
MGMT: *O*^6^-methylguanine-DNA methyltransferase
PFS: Progression-free survival
TMZ: Temozolomide

## References

[1] Yung WKA, Prados MD, Yaya-Tur R, Rosenfeld SS, Brada M, Friedman HS (1999). Multicenter phase II trial of temozolomide in patients with anaplastic astrocytoma or anaplastic oligoastrocytoma at first relapse. J Clin Oncol.

[2] Tatar Z, Thivat E, Planchat E, Gimbergues P, Gadea E, Abrial C (2013). Temozolomide and unusual indications: review of literature. Cancer Treat Rev.

[3] Baron M, Belin L, Cassoux N, Fardeau C, Blaizeau M, Soussain C (2020). Temozolomide is effective and well tolerated in patients with primary vitreoretinal lymphoma. Blood J Am Soc Hematol.

[4] Hart MG, Garside R, Rogers G, Stein K, Grant R. Temozolomide for high grade glioma. Cochrane Db Syst Rev. 2013. 10.1002/14651858.CD007415.pub2 PMC6457743 23633341

[5] Cohen MH, Johnson JR, Pazdur R (2005). Food and Drug Administration Drug approval summary: temozolomide plus radiation therapy for the treatment of newly diagnosed glioblastoma multiforme. Clin Cancer Res.

[6] Bae SH, Park M-J, Lee MM, Kim TM, Lee S-H, Cho SY (2014). Toxicity profile of temozolomide in the treatment of 300 malignant glioma patients in Korea. J Korean Med Sci.

[7] Mehta H, Gendle CS, Kumaran MS, Vinay K (2023). Temozolomide-induced drug rash with eosinophilia and systemic symptoms syndrome. Indian J Dermatol Venereol Leprol.

[8] Farshchian M, Bardhi R, Daveluy S (2021). Desquamative skin rash associated with temozolomide in a patient with glioblastoma. Dermatol Ther.

[9] Lee DE, Kaffenberger BH, Gru AA, Hamann D. Temozolomide-induced inflammation of disseminated superficial actinic porokeratosis. Dermatol Online J. 2018;24. 29634886

[10] Teitelbaum A. Eye symptoms and toxicities of systemic chemotherapy. MASCC Textbook Cancer Supportive Care Survivorship. 2010:333–47.

[11] Schmid KE, Kornek GV, Scheithauer W, Binder S (2006). Update on ocular complications of systemic cancer chemotherapy. Surv Ophthalmol.

[12] Al-Tweigeri T, Nabholtz J, Mackey JR (1996). Ocular toxicity and cancer chemotherapy: a review. Cancer: Interdisciplinary Int J Am Cancer Soc.

[13] Sakellakis M, Spathas N, Tsaousis KT, Nikitiadis EN, Linardou H, Diakonis VF. Potential ophthalmological side effects induced by anti-neoplastic regimens for the treatment of genitourinary cancers: a review. Cureus. 2022;14. 10.7759/cureus.27266 PMC9403378 36039252

[14] Gold JA, Shupack JL, Nemec MA (1989). Ocular side effects of the retinoids. Int J Dermatol.

[15] Paulose SA, Sherman SW, Glass LRD, Suh LH (2019). Dupilumab-associated blepharoconjunctivitis. Am J Ophthalmol Case Rep.

[16] Hegedus F, Mathew LM, Schwartz RA (2017). Radiation dermatitis: an overview. Int J Dermatol.

[17] Ianchenko SV, Sakhnov SN, Malyshev AV, Fedotova NV, OIu O, Grishchenko IV (2014). Treatment of chronic allergic blepharoconjunctivitis. Vestn Oftalmol.

